# Epidemiology of Kawasaki Disease in Asia, Europe, and the United States

**DOI:** 10.2188/jea.JE20110131

**Published:** 2012-03-05

**Authors:** Ritei Uehara, Ermias D. Belay

**Affiliations:** 1 Department of Public Health, Jichi Medical University, Shimotsuke, Japan; 2 Division of High-Consequence Pathogens and Pathology, National Center for Emerging and Zoonotic Infectious Diseases, Centers for Disease Control and Prevention, Atlanta, United States

**Keywords:** Kawasaki disease, incidence, seasonality, coronary artery, epidemiology

## Abstract

Kawasaki disease (KD) is a systemic vasculitis that mainly affects children younger than 5 years. Although Dr. Tomisaku Kawasaki first reported KD over 40 years ago, the cause of the disease remains unknown. Currently, KD has been diagnosed in more than 60 countries, including those in Asia, the Middle East, Latin America, and Africa, as well as in North America and Europe. The purpose of this review is to describe the epidemiologic features of KD—particularly its incidence, seasonality, and the occurrence of coronary artery abnormalities—primarily in Japan and the United States, but also in Europe and other Asian countries.

## 1. INTRODUCTION

Kawasaki disease (KD) is a systemic vasculitis that mainly affects children younger than 5 years. Although over 40 years have passed since Dr. Tomisaku Kawasaki first reported a case series of this disease,^1^ its cause remains unknown. Coronary artery abnormalities (CAAs), including dilatations and aneurysms, are the most serious complications of KD. Although KD is a self-limiting disease in most patients, CAAs develop in about 25% of untreated patients. Therefore, KD is recognized as a leading cause of acquired heart disease in children in developed countries.^2^

KD has been reported in many countries. In the United States, the first case series was described by Melish in 1976.^3^ Currently, more than 60 countries in Asia, the Middle East, the Americas, Africa, and Europe have reported KD cases.^4^ The purpose of this review is to describe the epidemiologic features of KD—with a focus on incidence, seasonality, and CAAs—in Japan and the United States, as well as in several European and other Asian countries. As this is not a systematic review, we cite the published literature on the descriptive epidemiology of KD. Because seasonal variation in KD is an important characteristic of descriptive epidemiology, and the seasonality of KD is unique, we have chosen to focus on the seasonality of KD.

### Japanese and American Heart Association guidelines

As the cause of KD is still unknown, it is defined based on its clinical manifestations. In Japan, the *Diagnostic Guidelines for Kawasaki Disease* (Fifth Revised Edition, February 2002) is widely used.^5^ The signs and symptoms can be classified into 2 categories: principal symptoms and other significant symptoms or findings. The principal symptoms are (1) fever persisting for 5 or days or longer (including fever that subsides before the fifth day in response to therapy), (2) bilateral conjunctival injection, (3) changes in the lips and oral cavity, (4) polymorphous exanthema, (5) changes in peripheral extremities, and (6) acute nonpurulent cervical lymphadenopathy. At least 5 of these 6 principal signs or symptoms should be present for a diagnosis of KD. However, KD can be diagnosed if patients with 4 of the principal signs or symptoms have a coronary artery aneurysm or dilatation, as determined by 2-dimensional echocardiography or coronary angiography. In the 2004 *American Heart Association Scientific Statement*, a fever persisting 5 days or longer is necessary for a diagnosis of KD.^2^ In other words, diagnosis of KD is based on a fever persisting 5 days or longer and the presence of at least 4 of the other 5 principal features. However, the diagnostic criteria defined by the American Heart Association are comparable to the Japanese diagnostic guidelines because 99% of patients with KD who were diagnosed using Japanese diagnostic guidelines also fulfilled the fever criterion.^6^ A diagnosis of incomplete or atypical KD can be made if echocardiographic evidence of CAAs and fewer than 4 principal features are present.

## 2. EPIDEMIOLOGIC FEATURES OF KAWASAKI DISEASE

### 2.1 Kawasaki disease incidence

#### Japan

In Japan, nationwide epidemiologic surveys of KD have been conducted almost every 2 years since 1970.^7^ The most recent survey with published data was conducted in January 2009 (the 20th survey) and included patients who visited hospitals during 2007–2008. The survey questionnaire and diagnostic guidelines were mailed to all specialized pediatric hospitals and general hospitals with a pediatric department having 100 or more beds. The survey method was similar throughout all the surveys.^8^ The total number of patients who visited hospitals during 2007–2008 was 23 337 (boys, 13 523 and girls, 9814), and the average annual KD incidence during the 2-year period was 216.9 per 100 000 children younger than 5 years (boys, 245.4 and girls, 187.0). The male-to-female ratio in incidence was 1.31. Among all reported patients during the 2-year period, 88.4% were younger than 5 years, and 67.7% were younger than 3 years. Age-specific incidence was highest among children aged 6 to 11 months and was very low among children aged 0 to 2 months. Among both boys and girls, incidence rate curves were unimodal. Three large epidemics were recorded in Japan—in 1979, 1982, and 1986. Although a similar nationwide epidemic has not been observed since 1990, the incidence of KD has more than doubled during the last 2 decades (Figure). The incidence in 2008 (218.6 per 100 000 children younger than 5 years) was the highest documented to date.

**Figure.**
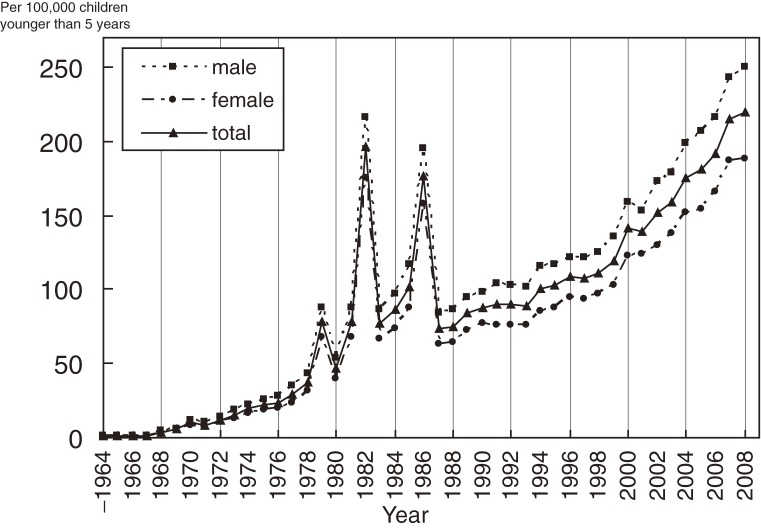
Trend in incidence rates of Kawasaki disease by sex in Japan

#### Korea

In Korea, nationwide surveys of KD have been conducted every 3 years since the 1990s.^9^ A questionnaire was sent to all hospitals that had a pediatric residency program. During 2006–2008, the average annual incidence of KD in Korea was 113.1 per 100 000 children younger than 5 years. This is the second highest incidence of KD in the world. KD incidence has also been increasing in Korea since 2000. The reason for the increasing trend in KD incidence in Japan and Korea is unknown.

#### Taiwan

An epidemiologic study of KD in Taiwan was conducted using the database of the National Health Insurance system, which covers most medical care costs of the Taiwanese population.^10^ During 2003–2006, the annual incidence of KD was 69 per 100 000 for children younger than 5 years in Taiwan. This rate was the third highest in the world. The male-to-female ratio was 1.62, which was higher than that in Japan.

#### China

In China, epidemiologic studies of KD were conducted in several provinces. In Beijing, a questionnaire and diagnostic guidelines were sent to target hospitals, and the method used to select the hospitals was similar to that used in Japan.^11^ A similar survey methodology was used in studies performed in Shanghai, and a slightly modified survey methodology was used in Sichuan province. In Beijing, the average annual incidence of KD during 2000–2004 was 49.4 per 100 000 children younger than 5 years. A significant increasing trend in KD incidence was observed during the 10-year study period from 1995–2004.

In Shanghai, a questionnaire and diagnostic guidelines were sent to 50 hospitals providing pediatric medical care.^12^ The average annual incidence during 1998–2002 was 27.3 per 100 000 children younger than 5 years.^13^ KD incidence increased to 46.3 per 100 000 children younger than 5 years from 2003–2007.

In Sichuan province, an epidemiologic study investigated patients hospitalized with KD in all hospitals in Sichuan province from 1997–2001.^14^ The incidence of KD was 7.1 per 100 000 children younger than 5 years during the study period. The incidence rate increased during each year of the study period.

In Hong Kong, a retrospective survey and prospective data collection were combined to assess the epidemiologic features of KD.^15^ The incidence in 1997–2000 was higher than that in 1994–1997 (39 vs 26 per 100 000 children younger than 5 years).

#### Thailand

A retrospective epidemiologic study of KD was conducted using the National Registry, which included data from all major cardiac referral centers in Thailand.^16^ The incidence of KD in 1998–2002 ranged from 2.12 to 3.43 per 100 000 children younger than 5 years.

#### India

An epidemiologic study was conducted using data from 1 tertiary hospital that provided services to all children in Chandigarh, North India.^17^ The incidence of KD was 0.51 per 100 000 children younger than 15 years in 1994. The incidence was calculated for children younger than 15 years because the median age of this study population was older. The incidence of KD showed an increasing trend during 1994–2008.

#### United States

In the United States, passive surveillance of KD has been conducted since 1976. Information on KD patients is collected using a standardized case report form that has been revised over the years. Although reporting of KD patients in the passive surveillance system is not complete, surveillance has allowed detection of nationwide KD outbreaks and monitoring of trends in cardiac complications, including CAAs. Monitoring trends in the occurrence of cardiac complications could help identify changes in diagnostic practices and the efficacy of intravenous immunoglobulin (IVIG) treatment.^18^ Passive KD surveillance is supplemented by periodic analysis of national hospital discharge records. The most recent analysis, which investigated US hospitalization data from 2009, showed a hospitalization rate for KD of 19 per 100 000 children younger than 5 years (unpublished data). Previous hospitalization rates per 100 000 children younger than 5 years were 20.8 in 2006, 19.6 in 2003, 17.1 in 2000, and 17.5 in 1997, as reported using data from the Kids Inpatient Database, the largest nationwide hospitalization database for children.^19^ Analysis of this database and other hospitalization databases revealed no clear evidence for a statistically significant increase in the incidence of KD in the United States for over 2 decades.^19^^,^^20^ In contrast, for reasons that are not well understood, a steady increase in KD incidence has been reported in many other countries, including Japan.

Studies in the United States have shown a clear variation in KD incidence by race and ethnicity. Incidence has consistently been highest among Asians and Pacific Islanders, followed in descending order by blacks, whites, and American Indians and Alaska Natives. As compared with whites, incidence is more than 2.5 times higher among Asians and Pacific Islanders and about 1.5 times higher among blacks. Although genetic or environmental factors could have a major role, the basis for these racial differences in KD incidence is poorly understood.

States with high Asian populations are expected to have a higher incidence of KD. Hawaii has the highest proportion of Asian and Pacific Islander populations and has the highest KD incidence of any US state. In 2010, approximately half of the 1.4 million residents of Hawaii were Asian, native Hawaiian, or other Pacific Islanders.^21^ KD incidence in Hawaii is about 2.5 times higher than that reported for the continental United States.^22^ During 1996–2006, among children younger than 5 years living in Hawaii, Japanese Americans had the highest KD incidence by far (210.5 per 100 000), followed by Native Hawaiians (86.9 per 100 000) and Chinese Americans (83.2 per 100 000). Children classified in the census as Other Asian (predominantly Korean Americans and Vietnamese Americans) had a KD incidence of 84.9 per 100 000. The incidence among Japanese-American children living in Hawaii was slightly higher than that for Japanese children living in Japan (184.6 per 100 000 for 2005–2006).^22^ This slight discrepancy may reflect differences in diagnostic practices and surveillance methodology. This high incidence among Japanese Americans relative to that of other Asian Americans living in Hawaii strongly indicates that genetic factors rather than environmental factors have a major role in the occurrence of KD among these populations. It appears that children of Japanese ancestry are at highest risk for KD regardless of their area of residence, ie, in Hawaii or Japan, which provides strong evidence for the hypothesis that genetic factors have a crucial role in KD occurrence. Similarly, KD incidence among whites living in Hawaii is remarkably similar to that among whites in the continental United States. In the continental United States, KD incidence differs by region: the northeast has the highest incidence, although the differences are not statistically significant.^19^

Many studies in the United States have indicated that 75% to 80% of KD patients are younger than 5 years and have a median age of approximately 1.5 years. KD incidence in boys is approximately 1.5 times that of girls. By age, 2-year-old children had the highest KD incidence (32.6), followed by those aged 1 year (23.1), 3 years (13.5), and 4 years (10.6).^19^ In 2006, KD resulted in an estimated $110 million in hospitalization costs in the United States.

#### Canada

Since the mid-1990s, periodic systematic surveillance for KD has been conducted in the province of Ontario, where approximately 39% of the Canadian population resides.^23^ KD incidence among children younger than 5 years was reported to have increased from 14.4 during 1995–1997 to 26.2 during 2004–2006. However, the incidence appears to have reached a plateau after 2000, which suggests that increased recognition of KD explains the rising trend in KD incidence from the mid-1990s to 2000. A recent study in Ontario reported a median age at onset of 3.3 years, which was higher than that reported in the United States.^23^

#### Europe

Several studies have estimated the incidence of KD in England. A doubling of KD incidence from 4.0 in 1991–1992 to 8.1 in 1999–2000 was reported in an analysis of hospital admission data.^24^ This doubling was partly attributed to increased recognition of the disease due to heightened awareness of the importance of early diagnosis and treatment.^24^ Most recently, analysis of hospital admissions data for England indicated that the KD incidence may have reached a plateau at around 8.4 per 100 000 children younger than 5 years.^25^ As expected, areas with high proportions of Chinese residents had the highest incidence of KD.

Studies in Scandinavian countries reported KD incidences ranging from 4.9 per 100 000 children younger than 5 years for Denmark (1999–2004),^26^ 6.2 for Sweden (1990–1992),^27^ and up to 7.2 for Finland (1982–1992).^28^ KD incidence in Denmark appears to have increased throughout the 1980s and 1990s, and stabilized after 1999. From June 1981 to March 1982, an outbreak of KD was recorded in Finland, where the annualized incidence more than quadrupled, reaching 31 per 100 000 children younger than 5 years.^29^

Elsewhere in Europe, a KD incidence of 15.2 per 100 000 children younger than 5 years was reported in Ireland for 1996–2000, which represents one of the highest reported incidences on the continent.^30^ For a 1-year period during 2005–2006, a KD incidence of 9 per 100 000 children was reported for northern France.^31^ In one of the earliest KD studies conducted in Europe, an incidence of 14.7 was reported in northern Italy during 1981–1982.^32^

The proportion of KD patients younger than 5 years ranged from 72% in a study in England to 77% in France. The median age ranged from 2 years in England and Ireland to 3 years in a study conducted in northern Italy. Boys consistently had a higher incidence of KD than girls. KD incidence rates are summarized in the Table.

**Table.** Reported incidence rates of Kawasaki disease in Asia, Europe, and North America

Region	Period	Incidence^a^	Data source	Citation (reference number)
**Asia**				
Japan	2008	218.6	Nationwide survey	Nakamura et al 2010 (8)
Korea	2006–2008	113.1	Nationwide survey	Park et al 2011 (9)
Taiwan	2003–2006	69.0	National Health Insurance database	Huang et al 2009 (10)
China				
Beijing	2004	55.1	Survey of hospitals in Beijing	Du et al 2007 (11)
Shanghai	2007	53.3	Survey of hospitals providing pediatric care	Ma et al 2010 (12)
Hong Kong	1997–2000	39.0	Retrospective survey and prospective data collection	Ng et al 2005 (15)
Sichuan province	2001	9.81	Survey of provincial hospitals	Li et al 2008 (14)
India	2007	4.5	Retrospective review of hospital records, Chandigarh, Northern India	Singh et al 2011 (17)
Thailand	2002	3.4	National Registry for Kawasaki Disease, including data from major cardiac centers	Durongpisitkul et al 2006 (16)
**North America**				
United States	2009	19.0	Hospitalization data (Kid’s Inpatient Database)	CDC, unpublished data
Hawaii	1996–2006	50.4	Hospital discharge records (Hawaii State Inpatient Database)	Holman et al 2010 (22)
Canada	2004–2006	26.2	Review of medical records from Ontario hospitals and cardiologists	Lin et al 2010 (23)
**Europe**				
England	1998–2003	8.4	Hospital admissions data	Harnden et al 2009 (25)
Ireland	1996–2000	15.2	Hospital discharge records (Ireland’s Hospital In-Patient Enquiry database)	Lynch et al 2003 (30)
Finland	1992	7.2	Active surveillance and hospital inpatient registry	Salo et al 1993 (28)
Denmark	1999–2004	4.9	Hospital discharge data (Danish National Hospital Register)	Fischer et al 2007 (26)
Sweden	1990–1992	6.2	Report from physicians and inquiries to all pediatrics and infectious disease departments	Schiller et al 1995 (27)
France	2005–2006	9.0	Prospective survey of pediatric departments in Northern France	Heuclin et al 2009 (31)
Italy	1981–1982	14.7	Medical record reviews and interviews with patient's family, Northeastern Italy	Tamburlini et al 1984 (32)

^a^Incidence rates were reported per 100 000 children <5 years of age, except for India, which was reported per 100 000 children <15 years of age.

### 2.2 Seasonality

#### Japan and Asia

In Japan, 2 seasonal peaks of KD incidence have been documented, in January (winter) and July (summer). The lowest number of KD patients was consistently reported in October (fall).

In Korea, where the second highest incidence of KD has been reported, seasonal occurrence of KD was observed in June and July (summer) and December and January (winter).^9^ The peak months of KD occurrence were May to June (summer) in Taiwan, whereas the lowest incidence was observed between November and January (winter).^10^ In China, 2 peaks of KD incidence were seen (in spring and summer) in Beijing, whereas the lowest incidence was observed in December and January.^11^ KD incidence peaks in May to August (summer and spring) in Shanghai.^12^ In Sichuan province, the highest incidence was observed in March through May, and the lowest incidence was in September.^14^ The peak occurrence of KD was seen in late spring and summer in Hong Kong.^15^

#### United States and Europe

In the United States, a seasonal peak in KD occurrence was observed in the winter and spring months. The winter peaks appear to be more prominent in the northeast and southern regions of the country.^18^^,^^19^ In contrast, no clear seasonal pattern was observed in Hawaii, owing perhaps to the tropical climate.^22^ A prominent seasonal increase in the occurrence of KD similar to that in the United States was reported in Ontario, Canada for winter but not spring.^23^ Analysis of hospital admissions data in England, Denmark, and Ireland indicated a similar peak occurrence of KD in the winter months.^25^^,^^26^^,^^30^ The seasonal peak in Finland was in both the autumn and winter months.^28^

### 2.3 Coronary artery abnormalities (CAAs)

#### Japan and Asia

CAAs are mainly detected by echocardiography.^33^ In Japan, the prevalences of coronary artery dilatation, aneurysm, and giant aneurysm (lumen size ≥8 mm) within 30 days after KD onset were 8.54%, 1.21%, and 0.25%, respectively, during the 2008–2009 study period. These proportions have been gradually decreasing since the end of the 1990s.^8^ Patients who are male, younger than 1 year, older than 5 years, or resistant to initial IVIG treatment have a higher risk of developing CAAs.^34^^–^^36^

In Korea, the proportions of coronary artery dilatation and aneurysms were 16.4% and 2.1%, respectively, during 2006–2008.^9^ The prevalence of CAAs during that period declined as compared with 2003–2005.^37^ The prevalence of coronary artery aneurysms (defined as ≥3 mm in diameter in children younger than 5 years or ≥4 mm in children older than 5 years) was 7.2% in Taiwan during 2003–2006.^10^ There was no significant decrease in the prevalence of coronary artery aneurysms during the 4-year study period. In Beijing, 20.6% of patients had CAAs, including dilatations and aneurysms.^11^ The development of CAAs showed a significant declining trend during 2000–2004. In Shanghai, the prevalences of coronary artery dilatation and aneurysms were 15.5% and 4.3%, respectively, during 2003–2007.^12^ In Sichuan province, 17% of patients with KD developed CAAs.^14^ The prevalence of CAAs was 8.5% in the fourth week after KD onset during 1994–2000 in Hong Kong.^15^ In Thailand, 14.5% of patients with KD who received IVIG treatment had CAAs.^16^

#### United States and Europe

In Ontario, Canada, during 2004–2006, approximately 4% of KD patients were reported to have developed coronary artery aneurysms, which was almost a 50% reduction from figures reported in the mid-1990s.^23^ In contrast, during a comparable time period in the United States, the proportion of KD patients with coronary artery aneurysms remained relatively stable at about 4%.^18^ An increase after 1999 in the proportion of KD patients with coronary artery dilatations observed in the United States was believed to be due to wider application of criteria developed by de Zorzi et al, which were published in 1998. The criteria were developed to improve diagnosis of dilatations by correlating coronary artery internal diameter with patient body surface area.^38^ Infants (younger than 1 year), older children (9–17 years), males, Asians and Pacific Islanders, and Hispanics have a higher risk of developing CAAs.^18^ Specifically, the rate for Asians and Pacific Islanders in the United States was higher than that for whites but similar to those reported in Japan, Korea, and China.

In European countries, the proportion of KD patients with CAAs reported were similar to that reported in North America, eg, 14% with dilatations and aneurysms in Sweden and 4.6% with aneurysms in Ireland.^27^^,^^30^ Slightly higher proportions of KD patients with CAAs were reported in recent studies in Northern France (18%) and Northern Italy (24%).^31^^,^^39^

## 3. PERSPECTIVES

Initially reported in the 1960s by Dr. Tomisaku Kawasaki as a distinct entity in the Japanese literature, KD was independently recognized as a new disease by Melish and Hicks in Hawaii in the early 1970s. Soon afterwards, descriptions of similar cases started to appear in the literature, including case descriptions from Canada (1975), Greece (1975), Australia (1976), West Germany (1977), and Belgium (1977). Patients with illness manifestations resembling KD were retrospectively identified as early as 1950 in Tokyo, Japan.^40^ The occurrence of such cases is believed to have been rare or nonexistent before 1950. Shibuya et al hypothesized that KD began in Japan after the Second World War, perhaps as a result of the introduction of an infectious agent.^40^ In contrast, in Western countries, some evidence indicates that children with illnesses similar to KD may have been identified since the late 19th century.^41^ Those cases may have been misdiagnosed as other childhood conditions such as infantile polyarteritis nodosa, Stevens Johnson syndrome, scarlet fever, and acute rheumatic fever. Although acute rheumatic fever was once a leading cause of acquired heart disease among children in developed countries, KD now holds that distinction in many of those countries. It is possible that KD has been increasingly unmasked by the disappearance of acute rheumatic fever in the post-antibiotic era.

In addition to possible genetic and environmental factors, the incidence of KD reported in different regions of the world can be affected by the survey/surveillance methods used, clinical diagnostic and treatment practices, physician awareness of KD, the data sources used to estimate incidence, the occurrence of KD clusters and outbreaks, and the population used to estimate incidence. Because nationwide epidemiologic surveys of KD have been conducted in a small number of countries, the results of those surveys were not always generalizable. In some countries, lack of KD awareness may distort the accuracy of reported incidences of KD. For instance, the reported KD incidences in Thailand and India are much lower than those in European countries, and KD was recently detected for the first time in Mongolia, in 9 patients.^42^ As awareness of this disease increases among physicians, incidence is expected to increase. Continuous monitoring of KD is critical to better understand the global extent of the disease.

Nationwide KD outbreaks have been documented in several countries, including Japan in 1979, 1982, and 1986, the United States in 1984–1985, Canada in the early 1980s, and Finland in 1981.^29^ Given the lack of knowledge regarding the etiology of KD, the factors that may have contributed to the occurrence of these outbreaks are poorly understood. Evidence from several countries shows that KD occurs in clusters and community-wide outbreaks. Population density and climatic factors have been shown to have a role in the occurrence of KD and might facilitate the occurrence of KD outbreaks. Taken together, these characteristics and the seasonal occurrence of KD indicate that the disease may be caused by an infectious agent or agents that remain elusive.

Disease susceptibility is thought to be an important factor in KD occurrence. The incidence of KD is high in Japan, Korea, and Taiwan, but it is low in North America and European countries. Asian ethnicity is a well-known risk factor for the occurrence of KD in Hawaii and the continental United States, indicating that genetic susceptibility may have a role in the occurrence of KD.

The introduction of IVIG as a mainstay treatment for KD has dramatically reduced the rate of CAAs in many countries. Thus, delayed diagnosis and treatment might result in a higher rate of CAAs. IVIG fails to ameliorate KD symptoms in up to 20% of KD patients. These patients are at a higher risk of developing cardiac complications and should be promptly retreated with a second dose of IVIG or other appropriate treatment to reduce the occurrence of cardiac complications. Identification of the causative agent of KD should facilitate prompt diagnosis and more specific treatment, leading to further reductions in morbidity and mortality. Additional studies should continue to focus on identifying the causative agent of KD, increasing understanding of the environmental and genetic factors that affect disease susceptibility, and, possibly, identifying disease markers that aid in early diagnosis of KD patients with incomplete or atypical presentation of the disease.
